# Paraneoplastic Neuro-Ophthalmologic Symptoms as Initial Manifestation of Hodgkin Lymphoma

**DOI:** 10.3390/hematolrep18010008

**Published:** 2026-01-05

**Authors:** Sophie-Charlott Seidenfaden, Thomas Graversgaard Adams, Peter Kamper, Sanne Jespersen, Martin Bjerregård Pedersen

**Affiliations:** 1 Department of Infectious Diseases, Aarhus University Hospital, Palle Juul-Jensens Boulevard 99, 8200 Aarhus, Denmark; soseid@rm.dk (S.-C.S.); sanne.jespersen@aarhus.rm.dk (S.J.); 2 Department of Ophthalmology, Aarhus University Hospital, Palle Juul-Jensens Boulevard 99, 8200 Aarhus, Denmark; thoada@rm.dk; 3 Department of Hematology, Aarhus University Hospital, Palle Juul-Jensens Boulevard 99, 8200 Aarhus, Denmark

**Keywords:** Hodgkin lymphoma, neuro-ophthalmologic symptoms, paraneoplasia, visual loss

## Abstract

**Background and Clinical Significance:** Patients with Hodgkin lymphoma (HL) often present with lymphadenopathy, biochemical inflammation, and constitutional symptoms, but may experience symptoms from extra-nodal organs. Symptoms are caused by either lymphoma or a paraneoplastic phenomenon but overt central nervous system (CNS) involvement in HL is very uncommon. However, in rare cases, paraneoplastic neuro-ophthalmologic manifestations occur. **Case Presentation:** This case report describes a young female diagnosed with HL initially presenting with visual loss, reduced visual field, impaired balance, and sensory disturbances but no evidence of CNS-lymphoma. After treatment with bleomycin, etoposide, adriamycin, cyclophosphamide, vincristine, procarbazine, and prednisolone (escalated BEACOPP), she experienced full recovery of all neurological and ophthalmological symptoms. She experienced complete remission without any signs of relapse at follow-up after 2.5 years. Paraneoplastic cerebellar degeneration (PCD) related to HL have been described as a rare neurological syndrome, with varying neurological symptoms preceding the diagnosis of HL. PCD is typically associated with anti-Tr antibodies. Despite being negative for anti-Tr antibodies in both serum and cerebrospinal fluid (CSF), the neuro-ophthalmologic symptoms were interpreted as a paraneoplastic phenomenon in HL resembling PCD. The exact pathophysiology in this case is unknown but might be associated with undetected antigens and T-cell-mediated autoimmunity because of the presence of non-malignant T-cells in the CSF. **Conclusions:** This manuscript describes a case of an atypical presentation of HL with neuro-ophthalmologic symptoms which fully recovered upon anti-lymphoma treatment. Because of the good prognosis, we aim to emphasize the awareness of rare cases of HL initially presenting such manifestations to avoid diagnostic delays.

## 1. Introduction

Classical Hodgkin lymphoma (HL) is a lymphoid malignancy characterized by few tumor cells (Reed–Sternberg cells) and a pronounced inflammatory microenvironment in tumor lesions. Most patients present with enlarged lymph nodes and systemic symptoms. HL may also affect extra-nodal tissues but direct lymphoma involvement of the central nervous system (CNS) is very uncommon. In some HL patients, symptoms may result from paraneoplastic processes. Isolated CNS and/or ophthalmologic symptoms as the first sign of HL is very uncommon and may therefore delay the diagnosis [1]. Ophthalmological symptoms in HL are even more scarcely reported [2]. Here, we report a case of neuro-ophthalmologic paraneoplastic symptoms with visual loss, reduced visual field, impaired balance, nystagmus, and sensory disturbances in a young female with newly diagnosed HL.

## 2. Case Presentation

A woman in her early twenties presented to the emergency department with a 4-week history of nausea, blurred vision, headache, sensory changes, fatigue, impaired balance, gait disturbance, and back pain. She had been diagnosed with an *E. coli* urinary tract infection five days earlier and treated with oral pivmecillinam. Her recent travel history included visits to Ecuador (Galápagos, freshwater lake), Rome, and London. Past medical history was notable for appendicitis and cruciate ligament rupture; no ophthalmological, neurological, or significant family history was reported. She did not use prescription medications or substances. The patient was stable with no constitutional symptoms. Notable findings included nautical dizziness, horizontal jerk nystagmus, and generalized paresthesia (excluding the face), along with bilateral positive Hoffmann and Babinski’s signs. Ophthalmological assessment showed low visual acuity (0.2 BCVA) in both eyes, anisocoria (left > right) with normal light responses, and no pathological changes upon slit lamp or fundoscopic exam. Labs revealed elevated WBC count (11.9 × 10^9^ /L), high CRP (79.9 mg/L), low hemoglobin (6.6 mg/dL), increased sedimentation rate (64 mm), high interleukin-2 receptor (3376 U/mL), and raised LDH (408 U/L). Serology indicated a past EBV infection with active reactivation (EBV-DNA 2090 copies IU/mL). Infectious screening was negative for other pathogens. CSF analysis found mononuclear pleocytosis (49 × 10^6^ /L), but normal protein and glucose, with further neuro-infectious workup reported in Table 1. Flow cytometry and morphologic evaluation of CSF showed that the mononuclear cells identified by biochemical analysis consisted of polyclonal T-cells, with a normal immune phenotype, no CD20-positive B-cells, or Reed–Sternberg cells. This finding was therefore interpreted as an inflammatory reaction involving the CNS. Additional testing of serum and CSF for paraneoplastic antibodies, including Anti-Tr, was also negative (see Table 1).

A thoracic X-ray showed widening of the mediastinum with prominent left hilum suspicious of hilar lymphadenopathy. Computed tomography (CT) and magnetic resonance imaging (MRI) of the cerebrum, brain stem, and medulla did not reveal any abnormal findings. However, a CT of the thorax, abdomen, and pelvis showed enlarged lymph nodes in mediastinum, parasternal, right hilar region, right axillary region, peri-clavicular, and in the apical right pleura. Accordingly, a flour-deoxy-glucose (FDG) positron emission tomography CT (PET/CT) concluded enlarged supra-diaphragmatic lymph nodes suspicious of lymphoma. Specialized ophthalmologic evaluation was performed, and a swept source optical coherence tomography demonstrated a bilateral thinning of the retinal ganglion cell layer but without pathologic changes to the retinal nerve fiber layer. Standard automated perimetry revealed visual field defects in a diffusely distributed pattern.

A biopsy, from an enlarged PET-positive lymph node on the neck, showed a pronounced inflammatory infiltrate with Reed–Sternberg cells (CD15+, CD30+, PAX5+ EBER+, CD20−) histo-morphologically corresponding to HL. The final diagnosis was, after diagnostic workup, therefore concluded to be EBV-positive classical HL, nodular sclerosis, and EORCT stage II with no B-symptoms, but an unfavorable prognostic profile, and neurological and ophthalmologic paraneoplastic symptoms, but with no evidence of CNS lymphoma involvement. At admission, prior to the final diagnosis, the patient was suspicious of neuro-infection and treated in a neuro-infection protocol consisting of intravenous acyclovir, penicillin, ceftriaxone, and dexamethasone until negative results rejected the diagnosis CNS infection. Fifteen days after initial admission and the diagnosis of HL was evident by biopsy, the patient started treatment with oral prednisolone 100 mg and allopurinol 300 mg daily. Based on the patient’s symptoms and the beneficial effect of the initiated pre-phase with corticosteroids, regular chemotherapy with bleomycin, etoposide, adriamycin, cyclophosphamide, vincristine, procarbazine, and prednisolone (BEACOPP) was initiated. An appropriate treatment plan was thoroughly discussed within the team for treating hematologists at the institution; despite being formally a stage IIA disease, the neurologic paraneoplastic symptoms were assigned to be indicative of advanced disease. Therefore, she started treatment according to advanced stage HL with BEACOPP escalated. Shortly after the first course of BEACOPP escalated, the patient had significant clinical improvement with near-complete recovery of all neurological symptoms. The symptoms started with sensory disturbances followed by visual loss and impaired balance, and seemed to resolve in the reverse order upon treatment. An interim evaluation with PET/CT, lumbar puncture, and ophthalmological examination was conducted after two courses of BEACOPP escalated. No intrathecal chemotherapy was administered at any time during the treatment. The PET/CT showed a complete metabolic remission (CR) with Deauville score 3, and CSF had a normal cell count; the visual loss also fully recovered with the patient regained normal vision and visual field. After four courses of BEACOPP escalated, a new PET-CT scan confirmed CR with Deauville score 2 and CSF was had normal results. Therefore, the treatment was stopped in accordance with national guidelines and the HD18 trial four months after index admission [3]. The patient is currently in an ongoing CR for HL at 2.5-years of follow-up and experienced full recovery of all ophthalmological and neurological symptoms. She has returned fully to daily activities and work without any after-care arrangements. Written informed consent has been obtained from the patient to publish this paper.

## 3. Discussion

This report describes a case of HL that presented with neuro-ophthalmologic symptoms. Mononuclear cells were identified in the CSF, without evidence of CNS lymphoma involvement. The patient received treatment based on an advanced stage HL protocol and experienced resolution of neurological symptoms, with ongoing complete remission of 2.5 years following the completion of therapy.

Ophthalmological symptoms are rare in HL. In a review by Valenzuela et al., only 30 cases of HL patients with ophthalmic manifestations were identified in the period of 1943–2021 and only 10 cases presented with ophthalmological manifestations prior to the diagnosis of HL [2]. The authors propose a classification of the eye manifestations in three groups: (i) direct infiltration, (ii) inflammatory reaction, or (iii) a paraneoplastic phenomenon, with inflammatory reactions [2]. To and colleagues reported a 24-year-old female who developed night blindness one week after initiation of chemotherapy for HL [4]. Retinal degeneration was suspected to be caused by an autoimmune process related to HL. The authors hypothesize that visual deficits were caused by an antibody cross-reaction to retinal proteins induced by chemotherapy [4]. In the current case, ophthalmological findings with profound visual deficits were interpreted as a paraneoplastic phenomenon and not related to prior chemotherapy. In contrast to the patient in the case from To et al. who suffered from progressive visual loss over a 10-year period, the patient presented here had visual loss prior to chemotherapy, and experienced full recovery of the ophthalmological symptoms during treatment. To and colleagues identified an autoantibody against retinal proteins in serum and proposed an association of the findings with this, but other antibodies may also be related to paraneoplastic retinopathies [4]. No retinal antibodies were detected in this current case report.

CNS symptoms are rare but occasionally seen in HL [1]. It can be caused by CNS lymphoma involvement or exist as a paraneoplastic phenomenon mediated by T-cell activation. The patient in this report was not diagnosed with primary cerebral HL or secondary involvement. The neurologic symptoms were instead suspicious of paraneoplastic cerebellar degeneration (PCD) related to HL.

PCD is described as a distinct rare paraneoplastic neurological syndrome, with a clinical presentation of varying cerebellar symptoms preceding the diagnosis of HL by months, and, in some cases, even years [5,6]. Patients suffering from PCD may present with unsteady gait, double vision, and impaired fine hand movements. Brain stem-related symptoms are also reported. Prodrome symptoms like nausea, vomiting, and flu-like illness are seen prior to the onset of deprived motor function, as seen in the present case report. Diagnostic workup of PCD in HL includes the detection of anti-Tr antibodies in serum and/or in CSF. Anti-Tr is an autoantibody directed against cerebellar Purkinje cells causing immune-mediated damage of the cells [7,8]. Other autoantibodies associated with PCD include anti-Yo, anti-Hu, ANNA1, ANNA2, and ANGA [9] and despite intensive testing, some cases of PCD can be seronegative [1]. This might be associated with undetected antigens or more T-cell-mediated underlying mechanisms in these cases. Antitumor therapy is the cornerstone in the treatment of PCD, and the symptoms usually resolve with treatment. This was also the case for the patient presented in this report. Her symptoms resolved in the reverse order they initially presented. Symptoms were indicative of indicating PCD, but no anti-Tr nor other paraneoplastic antibodies were detected in the diagnostic workup.

Ophthalmological symptoms have been reported as a component of the PCD with optic nerve affection. The patient in this report had a combination of both significant ophthalmological and neurological symptoms which is an uncommon presentation of HL. The patient was extensively and repeatedly investigated because of the progressive nature of the neurological symptoms prior to the diagnosis. This contributed to a relatively minor diagnostic delay, whereas others report a median diagnostic delay of 8 months (1–24 months) for PCD in HL.

The CSF pleocytosis with non-malignant T-cells was concluded to be of inflammatory origin [10]. Diagnostic diversity of CSF pleocytosis in 262 adult patients was investigated by Østergaard et al., revealing a distribution of 40.5% neuro-infections, 30.2% non-infectious neurological diseases, 8.8% malignancies, 7.6% infections outside the CNS, and 13% due to other conditions [10].

## 4. Conclusions

In conclusion, the combination of significant ophthalmological and neurological symptoms in HL is rare. This manuscript describes a case of an atypical presentation of HL with neuro-ophthalmologic symptoms which fully recovered upon anti-lymphoma treatment. Clinicians should be aware of cerebral paraneoplastic manifestations, including PCD, as an unusual first presentation of underlying malignancy, including lymphoma. Delayed diagnosis carries a risk of poorer outcomes in patients with otherwise favorable prognoses.

## Figures and Tables

**Table 1:** Selected microbiological and autoimmune screening performed as part of diagnostic workup. (P: plasma; S: serum; CSF: cerebrospinal fluid).

Microbiological and Paraneoplastic Screening	
S-Borrelia burgdorferi antibodies	IgG: Negative
S-Aspergillus galactomannan antigen	Negative
S-Toxoplasma gondii	IgM and IgG: Negative
S-Plasmodium DNA/RNA	Negative
S-Cytomegalovirus antibodies	IgM and IgG: Negative
S-Cytomegalovirus DNA/RNA	Negative
S-Epstein–Barr virus antibodies	IgM VCA: Negative IgG VCA: Positive EBNA: Positive
S-Epstein–Barr virus DNA	2090 copies IU/mL
P-Leptospira antibodies	Negative
P-Bartonella antigens	Negative
P-Wassermann antibodies	Negative
P-Syphilis serology screening	Negative
P-Schistosoma antibodies	Negative
P-Strongyloides DNA	Negative
P-Tenia antibodies	Negative
P-Aspergillus DNA	Negative
P-Galactomannan antigen	Negative
P-Histoplasma antibodies	Negative
CSF-Listeria monocytogenes/DNA	Negative
CSF-Hemolytic streptococci, type B/DNA	Negative
CSF-Staphylococcus aureus/DNA	Negative
CSF-Streptococcus pneumoniae/DNA	Negative
CSF-Herpes simplex virus 1+2/DNA	Negative
CSF-Varicella Zoster virus/DNA	Negative
CSF-Enterovirus/RNA	Negative
CSF-Parechovirus/RNA	Negative
CSF-Microscopy and cultures of the CSF	Negative
CSF-JC polyoma virus	Negative
CSF-Cytomegalovirus DNA/RNA	Negative
CSF-Epstein–Barr virus DNA	Negative
CSF-Human Herpes virus 6, 7 and 8	Negative
CSF-Adenovirus	Negative
CSF-Intrathecal Borrelia burgdorferi test	Negative
Paraneoplastic screening	
CSF-Cerebellum antibody IgG	Negative
CSF-Amphiphysin IgG	Negative
CSF-Dihydropyrimidinase-related protein 5-IgG	Negative
CSF-Glutamate decarboxylase IgG	Negative
CSF-Neuron nucleus Hu-IgG	Negative
CSF-Neuro-Oncological Ventral Antigen 1-IgG	Negative
CSF-Paraneoplastic Ma antigen family-like 2-IgG	Negative
CSF-Recoverin1-IgG	Negative
CSF-SOX-1 IgG	Negative
CSF-Titin-IgG	Negative
CSF-Zinc-finger protein ZIC 4-Ig	Negative
P-CDR2-IgG	Negative
P-DRP5-IgG	Negative
P-gad65-IgG	Negative
P-NOVA1-IgG	Negative
P-Tr-IgG	Negative
P-PNMA2-IgG	Negative
P-Recoverin-IgG	Negative
P-SOX-1-IgG	Negative
P-Titin-IgG	Negative

## Data Availability

Data is unavailable due to privacy and ethical restrictions.

## Abbreviations

The following abbreviations are used in this manuscript:

BEACOPP	bleomycin, etoposide, adriamycin/doxorubicin, cyclophosphamide, oncovin/vincristine, procabazine and prednisolone
CT	computed tomography
CSF	cerebrospinal fluid
EBV	Epstein–Barr virus
FDG	flour-deoxy-glucose
MR	magnetic resonance
PCD	paraneoplastic cerebellar degeneration
PCR	polymerase chain reaction
PET-CT	combined positron emission tomography and computed tomography scans
